# Proposal of recommended experimental protocols for *in vitro* and *in vivo* evaluation methods of boron agents for neutron capture therapy

**DOI:** 10.1093/jrr/rrad064

**Published:** 2023-09-16

**Authors:** Yoshihide Hattori, Tooru Andoh, Shinji Kawabata, Naonori Hu, Hiroyuki Michiue, Hiroyuki Nakamura, Takahiro Nomoto, Minoru Suzuki, Takushi Takata, Hiroki Tanaka, Tsubasa Watanabe, Koji Ono

**Affiliations:** Research Center for BNCT, Osaka Metropolitan University, 1-1 Gakuen-cho, Nakaku, Sakai 599-8531, Japan; Laboratory of Pharmaceutical Technology, Faculty of Pharmaceutical Sciences, Kobe Gakuin University, Kobe 650-8586, Japan; Department of Neurosurgery, Osaka Medical and Pharmaceutical University, 2-7 Daigaku-machi, Takatsuki-shi, Osaka 569-8686, Japan; Kansai BNCT Medical Center, Osaka Medical and Pharmaceutical University, 2-7 Daigaku-machi, Takatsuki-shi, Osaka 569-8686, Japan; Institute for Integrated Radiation and Nuclear Science, Kyoto University, 2, Asashiro-Nishi, Kumatori-cho, Sennan-gun 590-0494 Japan; Neutron Therapy Research Center, Okayama University, 2-5-1 Shikata-cho, Kita-ku, Okayama 700-8558, Japan; Laboratory for Chemistry and Life Science, Institute of Innovative Research, Tokyo Institute of Technology, 4259 Nagatsuta-cho, Midori-ku, Yokohama 226-8503, Japan; Department of Life Sciences, Graduate School of Arts and Sciences, The University of Tokyo, 3-8-1 Komaba, Meguro-ku, Tokyo 153-8902, Japan; Institute for Integrated Radiation and Nuclear Science, Kyoto University, 2, Asashiro-Nishi, Kumatori-cho, Sennan-gun 590-0494 Japan; Institute for Integrated Radiation and Nuclear Science, Kyoto University, 2, Asashiro-Nishi, Kumatori-cho, Sennan-gun 590-0494 Japan; Institute for Integrated Radiation and Nuclear Science, Kyoto University, 2, Asashiro-Nishi, Kumatori-cho, Sennan-gun 590-0494 Japan; Institute for Integrated Radiation and Nuclear Science, Kyoto University, 2, Asashiro-Nishi, Kumatori-cho, Sennan-gun 590-0494 Japan; Kansai BNCT Medical Center, Osaka Medical and Pharmaceutical University, 2-7 Daigaku-machi, Takatsuki-shi, Osaka 569-8686, Japan

**Keywords:** boron neutron capture therapy (BNCT), boron agents, evaluation protocols, *in vitro*, *in vivo*

## Abstract

Recently, boron neutron capture therapy (BNCT) has been attracting attention as a minimally invasive cancer treatment. In 2020, the accelerator-based BNCT with L-BPA (Borofalan) as its D-sorbitol complex (Steboronine®) for head and neck cancers was approved by Pharmaceutical and Medical Devices Agency for the first time in the world. As accelerator-based neutron generation techniques are being developed in various countries, the development of novel tumor-selective boron agents is becoming increasingly important and desired. The Japanese Society of Neutron Capture Therapy believes it is necessary to propose standard evaluation protocols at each stage in the development of boron agents for BNCT. This review summarizes recommended experimental protocols for *in vitro* and *in vivo* evaluation methods of boron agents for BNCT based on our experience with L-BPA approval.

## INTRODUCTION

**Fig. 1:**
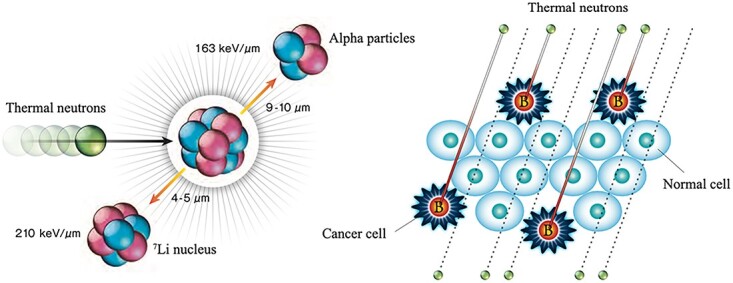
Neutron capture reaction of ^10^B (left) and a conceptual diagram of cancer cell-selective BNCT (right). Reproduced with permission from JSNCT website (http://www.jsnct.jp/e/about_nct/index.html).

Patient-friendly and minimally invasive therapies have recently gained popularity, allowing patients to return to their daily lives quickly. This is anticipated to lower healthcare costs while also assisting patients in realizing a healthy and fulfilling quality of life. In this regard, boron neutron capture therapy (BNCT) is attracting attention as a minimally invasive cancer treatment. In BNCT, the patients are given a boron agent and the target area is exposed to a neutron beam for several tens of minutes. In addition, compared with conventional radiotherapy, posttreatment skin damages are relatively small [1–3]. The thermal and epithermal neutrons used in BNCT have a very low energy of 0.025–10 keV, whereas the particles such as ^7^Li and ^4^He (α ray) nuclei produced by neutron capture reactions with boron (^10^B) have a high linear energy transfer with 210 and 163 keV/μm, respectively. Furthermore, their ranges inside tissue are 4–9 μm, so they come to a full stop inside the tissue where the reaction took place. There are other nuclei in the human body that can capture a thermal neutron. Hydrogen and nitrogen, in particular, are present in high concentrations and thus affect significantly on the neutron irradiation dose. Although the probability of a thermal neutron being captured by ^10^B atom is over 2000 times higher than the above-mentioned atoms, it is calculated that 10^9 10^B atoms per cancer cell are required to achieve cytocidal effects while minimizing the side effects. Thus, for BNCT, a cancer cell-selective radiotherapy, selective delivery of ^10^B to cancer cells is essential (Fig. 1). BNCT is effective for invasive cancers that are difficult to treat with conventional radiotherapy because thermal neutron irradiation can ideally generate α rays selectively to cancer cells.

In 2009, Japan succeeded in developing the world’s first accelerator to generate neutrons for BNCT. Until then, a nuclear reactor was the only source of neutrons for BNCT. The advent of accelerator neutron sources has made BNCT possible in hospitals, paving the way for general treatment [4, 5]. Finally, in 2020, the accelerator-based BNCT with L-BPA (Borofalan) as its D-sorbitol complex (Steboronine®) for head and neck cancers was approved by Pharmaceutical and Medical Devices Agency [6, 7].

Furthermore, neutron generation technology using accelerators is being developed in various countries [8–12]. Therefore, the development of novel tumor-selective boron agents is becoming increasingly important and desired. The Japanese Society of Neutron Capture Therapy (JSNCT) believes it is necessary to propose standard evaluation protocols at each stage in the development of boron agents for BNCT in the future. In this review, we summarize recommended experimental protocols for *in vitro* and *in vivo* evaluation methods of boron agents for BNCT based on our experience with L-BPA approval.

## PROPERTIES REQUIRED FOR BNCT BORON AGENTS

Conventional anticancer drugs are effective at concentrations in the order of 10^−6^–10^−9^ M in cancer cells because they often target cell growth signals or genes that are active in cancer cells. However, the drugs themselves do not always selectively accumulate and act on cancer cells. In BNCT, on the other hand, cells containing ^10^B are killed by thermal neutron irradiation, so the key point is how to accumulate ^10^B compounds in cancer cells. As stated beforehand, ~10^9 10^B atoms are required in the cancer cells to obtain sufficient therapeutic effect, which means that a boron concentration of about 10^−3^ M is required. As L-BPA is administered at 500 mg/kg in clinical practice [6,7], the development of boron agents used in BNCT requires a completely different approach from that of anticancer agents, which are usually effective at low doses. According to the biodistribution study in tumor bearing hamsters, the blood half-life of L-BPA is short, and boron concentrations in tumors are related to the blood concentration [13]. Therefore, in clinical practice, intravenous administration is continued during neutron irradiation to maintain the ^10^B concentrations in the blood [14]. It should be noted that, not all cancers can be treated with L-BPA, as some cancers do not take up L-BPA.

Successful treatment with BNCT is highly dependent on the ability of ^10^B to selectively accumulate in tumor cells and intracellular biodistribution. The following three characteristics are thought to be the ideal circumstances required for boron agents:

[1] The ^10^B concentration in tumor tissue should be kept constant throughout the neutron irradiation to maintain the expected antitumor effect (ideally 25~30 μg^10^B/g). The probability of a nucleus capturing a thermal neutron is expressed as a ‘thermal neutron capture cross section’ (barn = 10^−24^ cm^−2^). Elements in living organisms also capture thermal neutrons (e.g. H = 0.332 barns, *n* = 1.75 barns), but their thermal neutron capture cross sections are several orders of magnitude smaller than ^10^B (3838 barns). However, since these elements are present in high concentrations in the body, it cannot be ignored. It is calculated that, approximately 85% of the radiation dose is generated from ^10^B neutron capture reactions when the ^10^B concentration exceeds 25 μg/g [15].

[2] To ensure safety, the ratios of ‘concentration in tumor tissue/normal tissue’ and ‘concentration in tumor tissue/blood’ should be high (ideally >3) [16], and the systemic toxicity should be low.

[3] Boron agents should be rapidly excreted from normal tissues and blood after neutron irradiation. In other words, boron agents used for BNCT must have tumor selectivity to minimize effects on normal tissues and ^10^B concentration in tumor tissue to ensure a therapeutic dose to the tumor. Furthermore, boron agents should be considered for compliance with the International Council for Harmonization of Technical Requirements for Pharmaceuticals for Human Use (ICH) guidelines for ‘neoplastic agents’ in addition to national guidelines in each country. Needless to say, the criteria described here are based on the L-BPA approval.

## STANDARDIZATION OF EVALUATION METHODS FOR BORON AGENTS

### Boron concentration measurement methods

Inductively coupled plasma (ICP) or prompt gamma-ray analysis (PGA) is commonly used as a method to measure the boron concentration in blood, tissue, or *in vitro* samples. In this section, we briefly introduce these quantification methods. One point to note when using these methods to measure boron is that pretreatment should be performed without using laboratory equipment (e.g. borosilicate glass) that contains boron. For sample preparation, it is desirable to use boron-free plastic containers instead of glassware.

#### ICP-related analytical methods

**Fig. 2:**
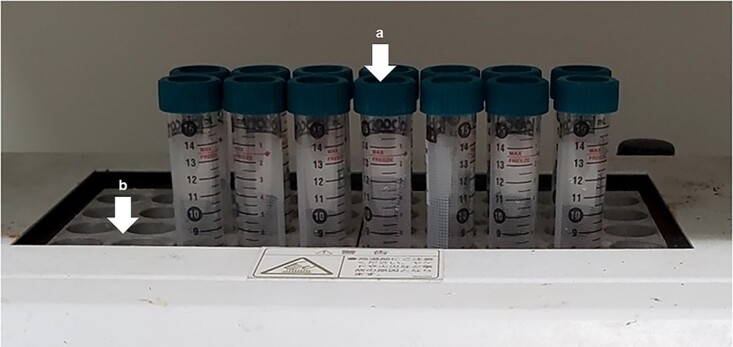
Wet-ashing procedure of boron-containing samples using dry block heating system. (**a**)Wet-ashing sample in centrifuge tube. (**b**) Dry block heater.

ICP is the gold standard for boron concentration determination. The main advantage of ICP is that it does not require neutron irradiation for measurement. ICP can be divided into two main categories: inductively coupled plasma atomic emission spectroscopy (ICP-AES) and inductively coupled plasma mass spectroscopy (ICP-MS). ICP-AES is sufficient for standard boron concentration measurement, while ICP-MS has excellent sensitivity and can discriminate between ^10^B and ^11^B. The measurement limit of ICP is ~0.1 ppm boron concentration. About 2–5 ml of solution is required for measurement. The sample is pretreated by being exposed to a strong acid at high temperature, a procedure known as wet ashing (Fig. 2). Removal of insoluble components in the sample is necessary before measurement. Due to the influence of the boron memory effect [17], after measuring a sample containing high concentrations of boron, the sample line should be thoroughly cleaned until the background value sufficiently decreases.

#### PGA method

PGA is a measurement technique for the analysis of ^10^B concentration and it involves measuring the prompt gamma rays emitted from the ^10^B(n, a)^7^Li* reaction [18]. PGA can be applied to any sample as long as it can be contained in a sample tube. The sample should be stored in a polytetrafluoroethylene (PTFE) container to reduce the generation of unwanted gamma rays. The detection limit of the system is 0.1–0.5 ppm ^10^B concentration. In practice, the ^10^B concentration inside a 1-g tissue sample can be measured in <30 s with 10% accuracy. Moreover, the system has the advantage that pretreatment of the sample is not required. To reduce the measurement uncertainty, the measurement time may be extended if the boron concentration in the sample is low.

#### Other methods

Analytical methods using enzyme-linked immuno sorbent assay (ELISA)[19] and fluorescence with molecular probes[20] are being investigated as methods for measuring the boron concentration.

These methods can detect lower concentrations in smaller amounts of samples compared with ICP-MS and/or PGA. However, the measurement of boron concentration by ELISA or fluorescence method using molecular probes is not yet a standard method. ELISA requires the use of antibodies to boron compounds [21], and these anti-boron compound antibodies are not commercially available. The fluorescence sensor method requires the use of molecular probes for boron compounds [22–25]. The fluorescence sensor for boron cluster compounds such as carborane, decaborane or dodecaborate is not yet reported; the sensor for boron compounds is only developed for compounds containing boric acid. Such methods will eventually be accepted as a standard and common method to determine boron concentration.

#### RBE or CBE

The biological effects of heavy particle radiation are different from those of X-rays due to the large LET. For the same physical dose of 1 Gy, the biological effect of 1 Gy of heavy particle radiation is larger than that of 1 Gy of X-rays. Therefore, when evaluating the effects of heavy particle radiation on tumors and normal tissues, the coefficient of conversion to X-ray equivalent dose, the relative biological effectiveness (RBE), is used to convert the dose.

The advantage of the RBE is that it can be used as a guide to the tolerable dose for each normal tissue, which has been empirically obtained with X-rays. In other words, the RBE is a tool to optimally and safely use heavy-ion beams, for treatment, by using the rule of thumb that states at what total dose (or higher a tumor effect) is observed or serious adverse events occur when converted to 2 Gy per dose of X-rays.

The concept of RBE applied to BNCT is the compound biological effectiveness (CBE). CBE values differ from one boron agent to another and from one tissue to another. Therefore, after the organ accumulation of a new boron drug is measured using the method described in this paper, it is recommended to evaluate the CBE of tumor tissue and normal tissue (organs at risk in radiotherapy) for that boron drug by performing animal experiments [26,27].

#### A recommended protocol for preparation of ICP samples

#####  

*Preparation of cell culture sample for measuring intracellular ^10^B concentration.* Cells were seeded and incubated. The medium was replaced with an equivalent amount of medium containing the boron compounds. After incorporation of the boron compound, the medium was removed by aspiration. The cells were washed thrice with ice-cold PBS(−), harvested by trypsinization and then counted. However, the washing conditions should be considered for each compound because low-molecular-weight compounds tend to be washed out. Trypsinization is performed with 0.05–0.25% trypsin at 37°C for 5–10 min. Trypsin is used at the lowest possible concentration to prevent drug efflux. When normalizing boron concentration per cell using protein amount per cell, collect about 1% of the cell suspension and measure the amount of protein in the cells by BCA (bicinchoninic acid) assay. Each sample was wet-ashed, and the boron concentration was measured by ICP-AES or ICP-MS [28].

#####  

*Selection of optimum wavelength.* When ashing blood samples, care should be made to prevent blowout and explosion, such as covering the sample with a lid before processing. When measuring boron concentration by ICP-AES, there are several candidate wavelengths, and the sensitivity and the effect of foreign substances in the sample differ depending on the wavelength used. For example, some boron wavelengths are interfered by tracer amounts of metals in the blood. Therefore, 208.9 nm is recommended for the measurement of boron concentration in blood to minimize the influence of these tracer amounts of metals. The user should select the optimal wavelength based on the samples and the experimental conditions.

### *In vitro* evaluation methods

The drugs for BNCT must deliver boron selectively to tumors without causing toxicity to normal cells. To evaluate the boron-containing drug for BNCT, cytotoxicity test and cellular uptake test for tumor cells are examined. To confirm the accuracy of these experiments, it is important to verify that the results obtained with conventional boron agents such as BPA and BSH are consistent with the results shown in previous studies. In particular, the usefulness of newly developed drugs can be clarified by comparing them with BPA, which has been proven to have a therapeutic effect. In these evaluations, it is important to clarify the physicochemical characteristics of the drugs in order to properly interpret the experimental results. For example, the high hydrophobicity of drugs is likely to cause aggregation at certain concentrations and compromise the intended function of the molecular design, leading to a risk of overlooking the advantage of the drugs. Thus, at the very least, the solubility of the drugs in water should be evaluated to properly interpret the obtained results. After the demonstration of these functions, BNCT effects with neutron irradiation are investigated if possible. Here, we describe the general methods of each experiment, their limitations and perspectives.

#### Preparation of compound solutions

It is preferable that sufficient water solubility of a drug is ensured before the *in vitro* evaluations. Exact solubility is not always necessary, but water solubility should be simply indicated with the following information: how much drug concentration was used to prepare an aqueous solution in *in vitro* studies or how much concentration was used to prepare a stock solution. It is also preferable to clarify that water solubility is guaranteed in the drug concentration range used in *in vitro* studies. If a stock solution is prepared with an organic solvent including DMSO, it is important to clearly describe how the stock solution was diluted with the aqueous solution for reproducibility.

#### Cytotoxicity

It is necessary to clarify the concentrations at which the drugs do not induce cytotoxicity in the absence of thermal neutrons. WST-1, WST-8 and MTT assays are often used to evaluate acute cytotoxicity. More specifically, many studies have reported the quantification of IC_50_ values or the comparison of cell viability at specific concentrations. Some drugs may show extremely low toxicity and it may be difficult to calculate the IC_50_ values. In such cases, a comparison of cell viability at specific concentrations should be useful to demonstrate that drugs do not cause unfavorable cytotoxicity in the following cellular uptake study.

#### Cellular uptake

This evaluation should be performed at the concentrations at which the drugs do not result in detectable cytotoxicity according to the study mentioned above. Many researchers use cells related to target disease or cells whose BPA uptake behavior has already been studied. It is recommended to use the same cell lines in *in vitro* and *in vivo* studies, which permits discussion about the correlation between *in vitro* and *in vivo* results.

Cellular uptake is usually indicated by the amount of boron, and it should be normalized by the number of cells, the amount of protein or the weight of the dried cells, because the amount of boron depends on the number of cells at the incineration process. Hence, the cellular uptake is often expressed as [μg boron/1 × 10^6^ cells], [μg boron/mg protein], [μg boron/mg cell], etc. In comparison with cellular uptake among multiple cell lines, it should be kept in mind that cellular uptake efficiency depends on many parameters including expression of transporters, the activity of endocytosis and so on.

Note that it has been reported that replacing the culture medium after administration of BPA with the medium containing low BPA resulted in the export of BPA from the cells, causing a decrease in intracellular boron concentration[29–31]. Thus, it is important to carefully examine the aforementioned procedures.

A recommended protocol of the boron uptake test is shown in “A recommended protocol for preparation of ICP samples” section.

#### Subcellular distribution

Since the range of α particles and Li recoil nuclei is within 10 μm, the intracellular localization of drugs is expected to induce higher BNCT effects than the extracellular localization. In addition, subcellular distribution of drugs is related to the cellular uptake mechanism. Thus, it is important to investigate the subcellular distribution. To visualize the distribution, previous studies utilized an antibody recognizing a boron compound, a molecular probe, a fluorescently labeled compound[32], NanoSIMS [33], etc. Specifically, subcellular BSH could be visualized by immunocytochemistry (ICC) with anti-BSH antibody [21,34], while BPA could be detected by the fluorescent probe that can react with the boronic acid moiety of BPA[35]. ICC staining using anti-BSH antibodies has been reported to evaluate the intracellular localization of drugs over time after intracellular transport of BSH by DDS or administration of BSH-binding compounds. Anti-BSH antibodies recognize both BSH alone and BSH-bonded peptides and can be used in the same manner as in conventional biological experiments [35].

#### Cellular uptake mechanism

Since BPA is mainly taken up via LAT1 amino acid transporter, which is overexpressed on many cancer cells, it can selectively be taken inside the tumor [36]. Meanwhile, some boron drugs have been reported to passively accumulate within tumors through diffusion[37]. Other drugs showed selective interaction with receptors or were taken up through endocytosis[38–40]. Clarifying such cellular uptake pathways will lead to the estimation of the selectivity of the therapeutic effect of BNCT. Thus, understanding the cellular uptake mechanism is essential to the development of drugs for BNCT.

To elucidate the mechanism, many studies have utilized inhibition assays with inhibitors or examined the effect of cell culture conditions such as oxygen and glucose concentrations on cellular uptake [41]. For example, in the case of BPA, it has been reported that the uptake amount is reduced by BCH, the inhibitor for system L transporter inhibitor [30,31]. If the uptake mechanism of a newly developed drug can be estimated, it is convenient to use a related inhibitor for a similar inhibition assay.

#### BNCT effects with neutron irradiation

To examine the BNCT effects of drugs in *in vitro*, thermal neutrons should be irradiated to the cells; however, in principle, cellular uptake is correlated with BNCT effects. Considering the limited availability of facilities for neutron irradiation (such as reactors and accelerators), it is not always necessary to examine *in vitro* BNCT effects if cellular uptake is quantitatively investigated.

It should be noted that biological experiments, such as cellular experiments, do not take rich ^10^B or natural boron compounds into account. This is because isotopes are biologically recognized as identical in cellular uptake and metabolism, and many experiments using isotope-labeled compounds have already been reported [42]. Needless to say, when considering BNCR by neutron irradiation, ^10^B-rich boron drugs are desirable.

#### Example of an experimental protocol to examine BNCT effects

**Fig. 3:**
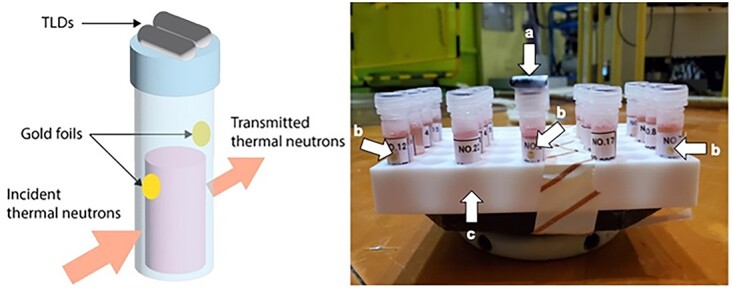
An example of sample preparation. (**a**) TLDs for measurement of gamma ray. (**b)** Gold foil for measurement of neutron fluence. (**c**) PTFE table.

The medium is replaced with a fresh medium containing boron compounds, followed by incubation. After removing the medium by aspiration, the cells are washed, trypsinized, collected and counted. Trypsin is then removed by aspiration after centrifugation, and the cells are suspended with the medium. As shown in Fig. 3, the cell suspension in the medium in a plastic vial is irradiated with thermal neutrons. The fluence can be determined by averaging the activation level of the two gold foils that are symmetrically attached to the surface of the plastic vial along the direction of incidence of the thermal neutrons. It should be noted that the vial should be made from a material that is insensitive to thermal neutrons. Although PTFE is the most suitable material for this purpose, polypropylene tubes that are widely used in biological experiments can also be used. Gamma rays that are not intended for BNCT can be determined by thermoluminescent dosimeters (TLDs) attached on the cap of the vial.

To evaluate the BNCT effect, the cell viability tests are examined such as colony forming test, WST assay or MTT assay using irradiated cells [35,43–45].

### *In vivo* evaluation methods

#### Acute toxicity test (single dose)

This section describes *in vivo* toxicity studies conducted as part of academic research for BNCT. In other words, the main objective of the study is to determine whether the planned dose of the new drug that is expected to have a medicinal effect is sufficiently safe. Single-dose toxicity is defined as a toxicological response that is indexed by a change in general condition, including lethality. When conducted as part of preclinical studies for clinical trials, it is necessary to conduct GLP-compliant studies in accordance with predefined guidelines. But when conducted as part of academic research, researchers should follow the GLP-defined guidelines (e.g. ICH-S4 toxicity studies) but can manage depending on the goal of their projects. When evaluating the antitumor effects and adverse events of new boron agents, it is always advisable to have a BPA + neutron irradiation group as a control group. In other words, the evaluation of the antitumor effects and adverse events of BNCT with novel boron agents should always be compared to the antitumor effects and adverse events of BNCT with BPA[46,47]. In *in vitro* studies, boron drug concentration in the medium is often mentioned in drug discovery research [48], but in *in vivo* studies, the boron drug dosage per mouse body weight is used for BNCT experiments [46]. It is desirable to convert the drug dose to the boron dose. The toxicity of the drug itself and the toxicity of the drug combined with neutron irradiation should be separately discussed from both perspectives.

#### Boron uptake study in tumors and various organs

After conducting the above-mentioned acute toxicity studies, *in vivo* pharmacokinetic studies are conducted. When performing *in vivo* pharmacokinetic studies, tumor-bearing animal models are often used to simultaneously evaluate the accumulation of the drugs in tumor tissues. PGA and ICP are commonly used to measure boron concentrations, and since PGA requires neutron irradiation [49], ICP has recently been used at the laboratory level [50].

The therapeutic effect ratio of BNCT depends on both the biological effect of the boron drug to each normal and tumor tissues evaluated by CBE and the boron accumulation ratio of tumor tissues compared to organs at risk(s). Therefore, it is common in the field of BNCT to evaluate the distribution of boron agents using the ratio of tumor tissue to normal tissue (T/N ratio) as well as the concentration of boron agents accumulating in each organ. However, in some tumor models such as brain tumors, the commonly used mouse subcutaneous tumor implantation model may have a different boron drug distribution compared to the orthotopic tumor model implanted in the tumor original lesion (e.g. brain in brain tumor model). Therefore, an orthotopic transplant tumor model is used as an ideal animal model to evaluate boron uptake [51]. Since the orthotopic tumor model are not always easy to perform, the allograft model (e.g. subcutaneous injection tumor model) is widely used for boron uptake study. Care must be taken for boron drugs targeting brain tumors, since the blood–brain barrier exists and the distribution of boron drugs may differ between the subcutaneous tumor model and the orthotopic tumor model [52].

#### The distribution study of boron agents in tissues

The microscopic distribution of the boron drug in the tissue (i.e. the distribution of boron) is a major determinant for the therapeutic effect of BNCT and the occurrence of adverse events. Therefore, it is necessary to evaluate the distribution of the boron drug in the tumor and normal tissues [13,46,53,54].

Various methods have been devised and the appropriate method largely depends on the type of drug. When a drug is labeled in advance with an evaluable atom or radioisotope, it is important to keep in mind that the properties of the drug may become modified. ^18^F-BPA, for example, is used to evaluate the pharmacokinetics of the BPA and its accumulation in tumor tissues before the application of BPA-based BNCT. However, the chemical properties of the drug change when ^18^F is bound to BPA; for example, the solubility of ^18^F in water is slightly different when ^18^F is bound to BPA [53]. Therefore, it must be rigorously examined whether the pharmacokinetics of ^18^F-BPA are really the same as those of BPA. To avoid possible changes in the function of the drug itself, radionuclide labeling, such as using ^14^C as the replacement of the drug constitutional atom, is preferred. However, there are not many laboratories that can use ^14^C during the drug discovery stage.

Alpha autoradiography (ARG) uses a special plastic detector named CR-39 to visualize the distribution of ^10^B atoms in a tissue and in a cell [55]. It is also applicable to every ^10^B-containing drug. However, the disadvantage is that it requires neutron irradiation.

The distribution of the boron agents BPA and BSH in tissue can also be examined by immunohistochemical staining using antibodies against these boron agents [56].

#### Evaluation of anti-tumor effects by neutron irradiation

Evaluation of antitumor effects with neutron irradiation is an important test for the development of boron drugs for BNCT. Such *in vivo* experiments are not defined as a GLP standard. To accurately evaluate the effect of BNCT on tumors, it is necessary to perform neutron irradiation only on tumors. A suitable shielding agent, such as a lithium fluoride plate [57], is used for this purpose. In this experiment, ^10^B-enriched drug compounds should be used to properly evaluate the therapeutic effect.

There are three main methods for evaluating effectiveness. The first is to remove the tumor after BNCT, isolate the cells by mechanical and enzymatic treatment and determine whether the cells are alive or dead by their ability to form colonies in the dish [58]. This is the most sophisticated radiobiological method. With this method, it is possible to determine the relationship between BNCT dose and effect, as in experiments with cultured cells. In other words, the effectiveness of BNCT can be evaluated by comparing the slopes with that of X-rays.

The second method uses the delay time (day) of tumor growth or the prolongation of animal survival time due to an inhibition of tumor growth as an indicator of BNCT effect. The tumor size can be evaluated by measuring the actual size of the subcutaneous tumor. Imaging methods are also used to evaluate the antitumor effects. One of the imaging methods is luminescence imaging [59,60]. To evaluate antitumor effect with this method, fluorescent proteins or enzymes such as luciferase should be induced to the tumor cells beforehand. In addition, diagnostic imaging methods such as CT and MRI can be used for the evaluation of the antitumor effects [61]. In environments where such imaging equipment is not available, direct observation in a time series with an appropriate control group is necessary for the evaluation.

The third method is to use TCD50 (50% radiation dose to control tumor growth) [62]. Using an *in vivo* carcinoma-bearing mouse model, the radiation dose to the tumor is progressively increased and the tumor control rate is experimentally determined. The TCD50 value is calculated as the value of 50% of the dose at which complete control of the tumor is obtained.

It should be noted that the handling of irradiated mice must conform to the radiation control regulations of each facility, since the standards differ from one country to another. When irradiating mice, the component of the neutron beam used in the neutron irradiation experiment should be recorded (For details regarding the component of neutron beam, please refer to section 6).

### Dosimetry for *in vitro*/*in vivo* experiments

Biological experiments for boron drug evaluation are usually carried out in a well-thermalized neutron irradiation field mixed with gamma rays and epithermal / fast neutrons. Radiation dose imparted to biological samples by charged particles emitted from ^10^B(n,α)^7^Li reactions, caused by thermal neutrons, is of the main interest. Besides that, in such irradiation fields, doses from ^14^N(n,p)^14^C reactions caused by thermal neutrons and ^1^H(n,n)^1^H reactions by higher energy neutrons should be considered as a background dose. Dose by gamma rays, which are mixed in the irradiation beam and generated by thermal neutron radiative capture reactions in the experimental equipment and the sample itself, is also involved. In this section, the determination methods for thermal neutron fluence, in addition to the background dose components, are described.

#### Determination of thermal neutron fluence

Biological response to boron drugs depends on the irradiated thermal neutron fluence as well as their intracellular or intratissue accumulation. Therefore, thermal neutron fluence is the most essential quantity to be determined as an index of irradiation dose in drug evaluation. Thermal neutron fluence at the target site can be experimentally determined by using the neutron activation method, which is the most common and reliable technique in dosimetry for BNCT. Small pieces of thin gold foils, which cause little perturbation of the irradiation field, are generally used for this purpose (gold activation). For instance, gold foils are attached to the surface of the cell containers or near the tumor transplantation sites of animals. After the irradiation, the thermal neutron fluence can be determined by measuring the radioactivity of the gold foils. However, gold also reacts to epithermal neutrons. So, to account for this, a correction factor which is experimentally determined during the characteristic evaluation of the irradiation field is necessary.

Thermal neutrons incident on a sample are scattered mainly by hydrogen atoms, resulting in an intensity gradient inside the volume of the target site. For instance, in the case of a parallel beam, attenuation of the neutron fluence occurs in the direction of the neutron beam. This nonuniform dose distribution inside the target site cannot be ignored in typical sized cell containers or transplanted tumors [63]. Consideration should be given to achieving a uniform distribution as much as possible by adopting procedures such as changing the location and/or direction of the samples during the irradiation when the nonuniformity may impair the analysis of the biological response [64]. If this is difficult to achieve, it is desirable to determine the volume-averaged thermal neutron fluence. For such a case, in the experiments using Kyoto University Reactor (KUR), gold foils are placed on both the beam entrance side and the exit side of the sample tube or the transplanted tumor to determine the average thermal neutron fluence. Furthermore, the gradient of the thermal neutron distribution becomes higher when irradiating samples with a large volume, such as stacking of sample tubes or microplates, and so on. In some cases, the gradient may be intentionally formed to have various dose conditions at once [65]. In such experiments, it is desirable to determine the thermal neutron fluence applied to each position of the samples as accurately as possible by increasing the number of measurement points.

#### Estimation of background doses

In the thermal neutron irradiation, gamma ray and neutron doses are always accompanied as the background dose that is independent of the accumulation of boron drugs. If these accompanying doses are not negligible in the analysis of biological effects, it is desirable to provide their levels as additional information to the thermal neutron fluence.

The gamma-ray dose can be approximated by measurement using a thermoluminescence dosimeter (TLD). Small TLDs, which cause little perturbation of the irradiation field, can be attached to the samples together with the gold foils. Glass dosimeters or optically stimulated luminescence dosimeters are also promising for this purpose. It should be noted that most of the commercially available gamma-ray dosimeters (including the TLD) have relatively high sensitivities to thermal neutrons. In the cases of KUR, a special quartz glass-enclosed BeO (Na) TLD with low sensitivity to thermal neutrons is used. Additionally, the contribution of thermal neutrons to the luminescence is corrected for by using the thermal neutron fluence measured using the gold foil [66]. External shielding cases may be considered to reduce the thermal neutron contribution; however, perturbation of the irradiation field should be carefully considered when using it simultaneously with the sample irradiation.

The background neutron dose is divided into thermal neutron dose and epithermal / fast neutron dose according to their energy ranges. The thermal neutron dose is mostly contributed from ^14^N(n,p)^14^C reactions due to nitrogen atoms in the sample. This can be derived by using the thermal neutron fluence, assuming the typical nitrogen content in the samples, as in the expression described below. The epithermal / fast neutron dose is generally difficult to measure, and *in situ* measurement methods such as the gold activation and TLDs have not been established. Usually, the dose is roughly evaluated based on the nominal value determined in characteristic measurement of the irradiation field. In the cases of KUR, the conversion coefficient from the thermal neutron fluence to the neutron dose is prepared based on the nominal neutron energy spectrum, and the dose in each experiment is quantified from the measured thermal neutron fluence [67].

#### Derivation of physical or equivalent dose

The use of physical absorbed dose as an index of irradiation dose is often useful for comparing the response to neutron irradiation directly with the control experiments using a reference radiation. The physical dose resulting from the ^10^B(n,α)^7^Li reaction due to thermal neutrons is derived by the following expression:


$$ {D}_{\mathrm{B}}\ \left[\mathrm{Gy}\right]=7.69\times{10}^{-14}\times \sqrt{\frac{0.0253}{kT\ \left[\mathrm{eV}\right]}}\times{C}_{\mathrm{B}}\left[\mathrm{\mu} \mathrm{g}/\mathrm{g}\right]\times \phi\ \left[{\mathrm{cm}}^{-2}\right], $$

where the numeric constant is the kerma coefficient of ^10^B for thermal neutron with the temperature of 0.0253 eV, calculated using the evaluated nuclear data file (ENDF) data library [68]. This value is modified according to the temperature $kT$ of the thermal neutron field, as in the second term [69]. The macroscopic ^10^B concentration is expressed as ${C}_{\mathrm{B}}$, where the measured value using the determination method such as ICP and PGA described in the previous section can be applied. $\phi$ is the thermal neutron fluence, where the measured value using the gold activation method can be used.

The thermal neutron dose from ^14^N(n,p)^14^C reactions is derived by the following expression:


$$ {D}_{\mathrm{N}}\ \left[\mathrm{Gy}\right]=6.98\times{10}^{-12}\times \sqrt{\frac{0.0253}{kT\ \left[\mathrm{eV}\right]}}\times \frac{N\ \left[\mathrm{wt}\%\right]}{100}\times \phi\ \left[{\mathrm{cm}}^{-2}\right], $$

where the numeric constant is the kerma coefficient of nitrogen for thermal neutron with the temperature of 0.0253 eV, calculated using the ENDF data library [68,70]. This value is modified according to the temperature $kT$, similarly to the above [70]. $\phi$ is the thermal neutron fluence. The weight fraction of nitrogen, expressed as $N$ typically in the range of a few percent, is usually assumed from typical nitrogen content of the subjects, such as a culture medium or a targeted tissue for *in vitro* or *in vivo* experiments, respectively.

The total physical dose is the sum of these doses and the epithermal / fast neutron dose ${D}_{\mathrm{f}}$ and the gamma-ray dose ${D}_{\mathrm{\gamma}}$, estimated by the above methods, as given in the following expression:


$$ {D}_{\mathrm{T}}={D}_{\mathrm{B}}+{D}_{\mathrm{N}}+{D}_{\mathrm{f}}+{D}_{\mathrm{\gamma}}. $$

Use of the equivalent dose has been often applied, especially to the analysis of *in vivo* experiments, in the cases that the biological response can be reasonably estimated against the neutron irradiation with/without boron drug administration, relative to a reference radiation [56]. The equivalent dose $ED$ is derived by using the following expression:


$$ ED=\mathrm{CBE}\times{D}_{\mathrm{B}}+{\mathrm{RBE}}_{\mathrm{N}}\times{D}_{\mathrm{N}}+{\mathrm{RBE}}_{\mathrm{f}}\times{D}_{\mathrm{f}}+{\mathrm{RBE}}_{\mathrm{\gamma}}\times{D}_{\mathrm{\gamma}}, $$

where the CBE is the boron CBE, the RBEs, the RBE of each dose component [71,72]. Typically, X-rays or gamma rays are selected as the reference radiation, and CBE and RBEs corresponding to them are used.

#### Additional consideration for reliable dosimetry

Toward establishing a more reproducible and stable dosimetry, it should be considered for similar experimental conditions to use a standardized irradiation geometry and to quantify the doses based on the well-validated dose characteristics [63,73]. Regarding the reproducibility of a measurement, for example, the results using a gold foil and a TLD may be affected by slight differences in the setup positions. In addition, the thermal neutron fluences, as well as the gamma-ray and neutron doses, in the different types of samples have different distributions depending on the shape and size of the samples. By setting up a standard geometry and defining accurate dose characteristics through measurements and well-validated simulation calculations, a reliable dosimetry protocol can be established for similar experiments. This may also help to improve the effectiveness of the measurement techniques used in each experiment. For instance, in addition to using a standard irradiation device, it should be considered for *in vitro* experiments to arrange the positioning, size and the number of tubes, and the amount of the medium, etc. to be the same for each irradiation, and for *in vivo* experiments to arrange the positioning and a number of animals to be the same for each irradiation, and adjust the size and location of the transplanted tumors uniformly as possible.

On the other hand, dosimetry by simulation calculation is a useful tool for dealing with various types of experiments and can lead to a more accurate evaluation in combination with an appropriate experimental validation. For example, it is necessary to consider the large dose gradient in the subject for *in vitro* experiments using relatively large dishes, flasks, well plates, etc., and for *in vivo* experiments using the animals with medium to large body size [64,70,71,74]. In such experiments, an accurate measurement is difficult or requires a great deal of effort. Simulation calculation can be quite effective for accurate dose estimation even in such a situation.

## SUMMARY AND FUTURE PROSPECTS

BNCT has gained worldwide attention since the turn of the 21st century, and new researchers are participating in the development of boron drugs. As a result, there are some data on the evaluation of the effects that employ unconventional methods. In addition, some of the data of researchers who have been involved in the past have not been correctly evaluated. Therefore, in this paper, we proposed to facilitate new drug development by conducting experiments and evaluations using a unified, standardized method.

First, standard methods were proposed for evaluating the uptake of boron compounds in cultured cells, evaluating the BNCT effect of neutron irradiation and searching for boron distribution. Furthermore, a method for the pharmacokinetics in experimental animals and a method for the evaluation of the effects of boron drugs in combination with neutron irradiation was proposed. In addition, standard methods and notations for the crucial dosimetry were proposed.

This proposal does not always demand a step-by-step evaluation of the effectiveness of the new drug from cultured cells to experimental animals and tumors. Drugs must be effective and safe in clinical BNCT. Although the efficacy of a drug in cultured cells is superior to that of the preceding drug, it is often inferior in the effects on animal tumors. To avoid such wastage, it is necessary to understand the mechanism of the cell-killing effect of the boron neutron capture reaction before proceeding with development. Considering that the cell-killing effect is due to high-LET particles with a range that does not exceed the cell diameter, the microscopic distribution of the drug and the morphological characteristics of the target cells are decisive factors in determining the effectiveness [75,76].

Although the finest distribution must be determined using antidrug antibodies or chemical probes, comparable precision can be achieved with ARG. ARG also has the advantage of being applicable to any drug containing ^10^B. A neutron source is required but can be provided by domestic research institutes. In the evaluation of effects, it is desirable to precede this microscopic distribution study in cells and tissues.

Since the distribution search can be applied not only to tumors but also to organs, it is very useful for predicting the extent of BNCT effects on normal tissues. After confirming the distribution, it is reasonable to investigate the effect and safety in cultured cells and experimental tumors.

As a rigorous evaluation of effects requires time, labor and money, it is preferable to precede the complete microdistribution study before the validation of effects. The evaluation in tumors should be examined in multiple steps of neutron dose or drug dose. This is because, when compared with previous drugs, a favorable result may be obtained at one level, but in an increased dose of neutron or drug, there may be no difference in the effects.

Since the proposals in this paper are standard at present, we expect a simpler and more useful methods will be developed in the future.
