# Correction to: Proposal of recommended experimental protocols for *in vitro* and *in vivo* evaluation methods of boron agents for neutron capture therapy

**DOI:** 10.1093/jrr/rrad078

**Published:** 2023-09-29

This is a correction to: Yoshihide Hattori, Tooru Andoh, Shinji Kawabata, Naonori Hu, Hiroyuki Michiue, Hiroyuki Nakamura, Takahiro Nomoto, Minoru Suzuki, Takushi Takata, Hiroki Tanaka, Tsubasa Watanabe, Koji Ono, Proposal of recommended experimental protocols for *in vitro* and *in vivo* evaluation methods of boron agents for neutron capture therapy, *Journal of Radiation Research*, 2023;, rrad064, https://doi.org/10.1093/jrr/rrad064

In the originally published version of this manuscript, the following sentence was omitted from the Acknowledgements section:

‘This work was conducted as part of the activities of JSNCT.’

This has been corrected.

